# Effects of Different Organic Manures on the Biochemical and Microbial Characteristics of Albic Paddy Soil in a Short-Term Experiment

**DOI:** 10.1371/journal.pone.0124096

**Published:** 2015-04-16

**Authors:** Qian Zhang, Wei Zhou, Guoqing Liang, Xiubin Wang, Jingwen Sun, Ping He, Lujiu Li

**Affiliations:** 1 Ministry of Agriculture Key Laboratory of Plant Nutrition and Fertilization, Institute of Agricultural Resources and Regional Planning, Chinese Academy of Agricultural Sciences, Beijing, P.R. China; 2 Institute of Soil and Fertilizer, Anhui Academy of Agricultural Sciences, Hefei, P.R. China; University of Porto, PORTUGAL

## Abstract

This study aimed to evaluate the effects of chemical fertilizer (NPK), NPK with livestock manure (NPK+M), NPK with straw (NPK+S), and NPK with green manure (NPK+G) on soil enzyme activities and microbial characteristics of albic paddy soil, which is a typical soil with low productivity in China. The responses of extracellular enzyme activities and the microbial community diversity (determined by phospholipid fatty acid analysis [PLFA] and denaturing gradient gel electrophoresis [DGGE]) were measured. The results showed that NPK+M and NPK+S significantly increased rice yield, with NPK+M being approximately 24% greater than NPK. The NPK+M significantly increased soil organic carbon (SOC) and available phosphate (P) and enhanced phosphatase, β-cellobiosidase, L-leucine aminopeptidase and urease activities. The NPK+S significantly increased SOC and available potassium (K) and significantly enhanced N-acetyl-glucosamidase, β-xylosidase, urease, and phenol oxidase activities. The NPK+G significantly improved total nitrogen (N), ammonium N, available P, and N-acetyl-glucosamidase activity. The PLFA biomass was highest under NPK+S, followed by NPK+M and NPK+G treatments. Principal component analysis (PCA) of the PLFA indicated that soils with NPK+M and NPK+S contained higher proportions of unsaturated and cyclopropane fatty acids (biomarkers of fungi and gram-negative bacteria) and soil under NPK+G contained more straight chain saturated fatty acids (representing gram-positive bacteria). PCA of the DGGE patterns showed that organic amendments had a greater influence on fungal community. Cluster analysis of fungal DGGE patterns revealed that NPK+G was clearly separated. Meanwhile, the bacterial community of NPK+M treatment was the most distinct. RDA analysis revealed changes of microbial community composition mostly depended on β-xylosidase, β-cellobiosidase activities, total N and available K contents. The abundances of gram-negative bacterial and fungal PLFAs probably effective in improving fertility of low-yield albic paddy soil because of their significant influence on DGGE profile.

## Introduction

Fertilization is an important agricultural measure that affects soil quality and sustainable utilization of soils. Low inputs of organic materials and excessive use of mineral fertilizers have contributed to a general reduction in soil organic carbon (SOC) contents, with a consequent decline in agricultural soil quality. The appropriate utilization of manures and/or crop residues within management systems can increase levels of plant nutrients and enhance soil microbial biomass, activity and diversity [1, 2]. A considerable number of studies have focused on the effects of organic manures on soil physicochemical properties, such as SOC [3, 4]. However, increases in the SOC content following the addition of organic matter may take considerable time. Consequently, changes in SOC cannot fully and sensitively reflect the influence that the complexity of the organic compounds may have on the microbiological processes controlling nutrient availability. To make up for these defects, other soil indicators to study the effects of organic manure applications have been proposed [5]. The biochemical parameters include variables directly related to microbial activity (microbial biomass carbon, soil respiration etc.) [6], and extracellular enzymes involved in the carbon (C), nitrogen (N) and phosphorus (P) cycles in soil, which are more sensitive to environmental stress, play a major role in the degradation of organic matter, and provide rapid and accurate information on soil quality [5]. Most organic manures added into soil contain polymeric compounds, and thus the decomposition of these organic matters depends on the microbial production of extracellular enzymes and their break down should occur before taking up of low molecular weight organic molecules by microbial cells [7, 8, 9].

Because the growth and activity of microorganisms are sensitive functions of soil properties including nutrition, texture, pH, temperature, and water content, dynamic changes of microbial community can represent the improving effects of different types and amounts of organic materials on soil quality [10, 11]. A variety of microbial parameters have the potential for use as indicators of soil quality such as microbial biomass and microbial diversity [12, 13, 14]. The determination of phospholipid fatty acid analysis (PLFA) profiles reveals the structural characteristics of the microbial community and also provides estimate of the abundance about various microbial groups [15, 16]. The microbial community, size and activity showed by PLFA vary with different fertilizer managements, and therefore affect soil fertility and productivity [17]. Increases in the fungi/bacteria have been linked to increases in soil C and ecological buffering capacity [18, 19] and in response to organic management [19] as well as various organic amendments, such as livestock manure [20], crop residue [21] and green manure [22]. Other studies have shown increases in PLFA biomarkers for arbuscular mycorrhizal fungi (AMF) in response to composted green waste as well as long-term organic management [23, 24, 25]. Analysis with PCR-DGGE has been used to isolate microbial DNA and identify predominant microbial populations in a variety of systems to reflect the genetic diversity of the microbial community [26, 27]. There is an abundance of studies about the effects of different fertilizations on rice yield and soil fertility [28, 29, 30]. Changes under different tillage managements and different fertilizer regimes and sampling times have been investigated by the combination of PLFA and DGGE profiles [31, 32] which reflect both the community and genetic diversity of microorganisms.

However, direct comparisons between different organic manures have not been fully explored by the combination of PLFA and DGGE profiles, especially under albic paddy soil which is a typical low-yield soil in the Anhui and Jiangsu provinces of China and covers an area of 1 million hectares. Because of rain wash, loss of clay particles and deposition of silt and sand particles, albic paddy soil has an obvious condition of poor nutrition, a shallow plough layer and bad structure [33]. Long-term fertilizer experiment of quaternary red clay showed that most microbial parameters were mainly correlated with SOC and N, P which did not directly affect microbial parameters but did so indirectly by increasing crop yields, thus promoting the accumulation of soil organic matter [34, 35]. Experiment under a typic haplustoll silt loam in Argentina showed total PLFA content was lowest in soil under reduced tillage and soybeen monoculture and DGGE analysis estimated fungal communities were influenced by combined effects of crop rotation and tillage system [31].

So far, organic amendments to albic soil have focused on returning straw as well as livestock manure and green manure additions, of which single measures are preferred. The influence of organic matter on soil physical properties depends upon the amount, type and size of the added organic materials [36]. To explore suitable measures for improving albic paddy soil, three different organic manure carbon sources (livestock manure, straw and green manure) were investigated. Eight extracellular enzyme activities and PLFA and PCR-DGGE profiles were determined to evaluate the effects of different organic manures on extracellular enzyme activities and the microbial community and genetic diversity in a low yield albic paddy soil. The results would explore changes of soil fertility under different organic manure amendments by measuring soil biochemical and microbiological properties and thus provide strong evidence for improving the quality of low productivity albic paddy soils.

## Materials and Methods

### Field design and sampling

The study was conducted in Changfeng (32°22′58″N, 117°8′43″E) in Anhui province of east China, across the Huai River and the north-south Yangtze River. The site is located in the warm temperate and subtropical transition zone and has a warm humid climate with an annual average temperature and precipitation of 16.0°C and 1070 mm, respectively. Furthermore, this study was carried out on private land and the owner of the land gave permission to conduct the study on this site on the basis of a certain rent. This work was approved by Institute of Soil and Fertilizer, Anhui Academy of Agricultural Sciences who is in charge of the communication with the owners. At the start of the experiment, the soil had a pH (H_2_O) of 5.68, 6.32 g kg^-1^ organic carbon (C), 0.68 g kg^-1^ total nitrogen (N), and 5.17 and 96.1 mg kg^-1^ of available phosphorus (P) and potassium (K), respectively. The experiment was a wheat-rice rotation in a completely randomized design of four treatments with three replicates in 20 m^2^ plots. The four treatments were investigated as follows:

1. Chemical fertilizer (NPK) corresponding to180 kg ha^-1^ N, 90 kg ha^-1^ P_2_O_5_ and 120 kg ha^-1^ K_2_O.
2. Extra livestock manure addition (NPK+M), 22500 kg ha^-1^.
3. Extra wheat straw addition (NPK+S), 3000 kg ha^-1^.
4. Extra green manure addition (NPK+G), 22500 kg ha^-1^.

The amounts of organic manures applied were based on the farmers' practice and recommendation from the local academy of agricultural sciences. The chemical fertilizer type used and their nutrient contents are explained as follows: urea (N: 46%), (NH_4_)_2_HPO_4_ (N: 18%, P_2_O_5_: 46%), KCl (K_2_O: 60%). The livestock manure had 5.6, 4.2 and 3.3 g kg^-1^ total N, P and K, respectively, ~80% water content and 27.2:1 C:N. The straw had 3.2, 2.7 and 5.6 g kg^-1^ total N, P and K, respectively, ~15% water content and 84.8:1 C:N. The green manure had 5.4, 0.7 and 3.0 g kg^-1^ total N, P and K, respectively, ~65% water content and 16.1:1 C:N. The C content of three organic manures was approximately equal. Organic manures, P and K fertilizers were applied as basal fertilizers, while 70% of the N was applied as a basal dressing and 30% top-dressed on the rice ~20 days after transplanting at rice tillering stage. Seeds were sown on May 10, 2011, transplanting was June 13 and harvest was September 28. Rice planting density and cultivation management was consistent with local farmers. Soil samples (0–20 cm), consisting of five cores, were randomly collected from every plot on September 22, 2011 at harvest stage, when the activities of soil microorganisms were relatively stable. The five sub-samples were mixed into one sample, representing each replicate of the four treatments. The samples were immediately transported to the laboratory. Plants roots were removed by 2 mm sieving, and the samples were then stored at room temperature for chemical analysis, at 4°C for extracellular enzyme analysis, at −20°C for PCR-DGGE and at −80°C for PLFA analysis (the soil was freeze-dried before the determination of PLFAs).

### Chemical analysis

Soil pH was measured with a compound electrode (PB-10, Sartorius, Germany) using a soil to water ratio of 1:2.5 [17]. Soil organic C was determined by dichromate oxidation [37] and total N by Kjeldahl digestion [38]. Ammonium N (NH_4_^+^-N) and nitrate N (NO_3_^-^-N) contents were extracted with 1 M KCl solution (dry soil: KCl = 1:5) for 1 h [39], and NH_4_^+^-N and NO_3_^-^-N concentrations were determined by flow injection autoanalyzer (FLA star 5000 Analyzer, Foss, Denmark). Available P was determined by the Olsen method [40] and available K was analyzed by ammonium displacement of the exchangeable cations [11].

### Extracellular enzyme activities

The activities of all tested extracellular enzymes except urease and phenol oxidase were measured using MUB-linked or AMC-linked model substrates yielding the highly fluorescent cleavage products 4-methylumbelliferyl (MUB) or 7-amino-4-methylcoumarin (AMC) upon hydrolysis [41, 42, 43] (Table 1). The method is very sensitive and allowed a high throughput analysis of enzymatic activities [43]. Specifically, each equivalent of 1.0 g dry mass of fresh soil was added into a 100 mL centrifuge tube, and it was homogenized with 50 mL of 50 mM acetate buffer using a polytron homogenizer, then the mixture was poured into a round wide-mouth beaker. An additional 50 mL of acetate buffer washed the centrifuge tube and was poured into the same beaker. A magnetic stirrer was used to maintain a uniform suspension. The buffer, sample suspension, 10 μM references and 200 μM substrates (Table 1) were dispensed into the wells of a black 96-well microplate according to the strict volume and order described by Deforest [41]. The microplates were covered and incubated in the dark at 25°C for 4 h and the fluorescence quantified using a microplate fluorometer (Scientific Fluoroskan Ascent FL, Thermo) with 365 nm excitation and 450 nm emission filters [42]. The activities were expressed in units of nmol h^-1^g^-1^.

**Table 1:** Extracellular enzymes assayed in the sampled soil, their enzyme commission number (EC) and corresponding substrate (L-DOPA = L-3, 4-dihydroxyphenylalanine, 4-MUB = 4-methylumbelliferyl).

Enzyme	Substrate	EC
Phosphatase	4-MUB-phosphate	3.1.3.1
β-glucosidase	4-MUB-β-D-glucoside	3.2.1.21
β-cellobiosidase	4-MUB-β-D-cellobioside	3.2.1.91
N-Acetyl-glucosaminidase	4-MUB-N-acetyl-β-D-glucosaminide	3.2.1.30
β-xylosidase	4-MUB-β-D-xyloside	3.2.1.37
L-leucine aminopeptidase	L-Leucine-7-amino-4-methylcoumarin	3.4.11.1
Phenol oxidase	L-DOPA	1.10.3.2

The non-fluorometric enzyme, phenol oxidase, was measured spectrophotometrically in the clear 96-well microplate using the substrate of L-3, 4-dihydroxyphenylalanine (L-DOPA). The dispensed volume and the order of buffer, sample suspension, 25 mM L-DOPA and 0.3% H_2_O_2_ were the same as for the fluorometric enzymes [42]. The microplates were covered and incubated in the dark at 25°C for 20 h, and the activities were assayed by measuring the absorbance at 450 nm using the microplate spectrophotometer and expressed in unites of μmol h^-1^g^-1^. Urease activities was determined using urea as the substrate as described by Lu [44]. The determination was based on the product of ammonium, which was determined by kjeldahl apparatus. The activities were expressed as milligram of ammonium released per kilogram of soil per hour (ω (N) mg NH_4_^+^-N/kg/h).

### PLFA profiles

Differences in the microbial community and microbial biomass among the various nutrients managements were determined by phospholipid fatty acid (PLFA) analysis following the procedure described by Wu et al. [45]. Briefly, three-gram freeze-dried soil samples were used to extract the PLFAs with a single-phase mixture of chloroform: methanol: citrate buffer (15.2 mL at a 1:2:0.8 volume ratio). The extracted fatty acids in the chloroform were fractionated into neutral lipids, glycolipids, and polar lipids using a silica-bonded phase column (SPE-Si, Supelco, Poole, UK) with chloroform, acetone and methanol, respectively. The recovered polar lipids were transesterified to the fatty acid methyl esters (FAMES) by a mild alkaline methanolysis. FAMES were quantified by gas chromatograph (N6890, Agilent) and identified with an MIDI SHERLOCKS microbial identification system (Version 4.5, MIDI, Inc., Newark, DE). Nonadecanoic acid methyl ester (19:0) was added as the internal standard. Concentrations of PLFAs were expressed in units of nmol g^-1^.

Total microbial biomass and the abundance of individual PLFAs were determined using the total concentration of PLFAs (nmol g^-1^). PLFAs were divided into various taxonomic groups based on previously published PLFA biomarker data [23, 46, 47, 48]. Specifically, 14:0,15:0, 16:0, 17:0, 16:1ω5c, 16:1ω7c, 16:1ω9c, 17:1ω8c, 18:1ω5c, 18:1ω7c, a15:0, a17:0, cy17:0, cy19:0ω8c, i14:0, i15:0, i16:0, i17:0 and i19:0 were used to represent bacterial biomarkers. The polyunsaturated PLFAs 18:2ω6,9c, 18:1ω9c and 18:3ω6c(6,9,12) [49] were chosen to indicate fungal biomarkers. The fatty acids 16:0 (10Me), 17:0 (10Me) and 18:0 (10Me) were considered the biomarkers of actinomycetes, and 14:0, 17:0, 18:0, i14:0, i15:0, i16:0, i17:0, a15:0, a17:0 and cy17:0, cy19:0ω8c, 16:1ω5c, 16:1ω9c, 16:1ω7c, 17:1ω8c, 18:1ω5c, 18:1ω7c were considered to be gram-positive and gram-negative bacteria biomarkers, respectively.

### DNA extraction, PCR amplification and DGGE analysis

Soil total DNA was extracted from 0.5 g fresh soil using a Fast DNA SPIN Kit for soil (MP Biomedicals, Illkirch, France) according to the manufacturer’s instructions. DNA was finally eluted with 100 μL of the DNA elution solution included in the kit. Successful DNA extraction was characterized by electrophoresis on 1% (wt/vol) agarose gels.

The forward primer with GC clamp 357F-GC (5’-CGCCCGCCGCGCGCGGCGGGCGGGGCGGGGGCACGGGGGGCCTACGGGAGGCAGCAG-3’) and reverse primer 518R (5’-ATTACCGCGGCTGCTGG-3’) were used for bacterial PCR amplification targeting the V3 hypervariable region. Fungal genes were amplified using primers ITS1-F (5’-CTTGGTCATTTAGAGGAAGTAA-3’) and ITS2 (5’-GCTGCGTTCTTCATCGATGC-3’) [50, 51]. The concentrations of bacterial and fungal primers were 10 μM and 20 μM, respectively. PCR mixtures consisted of 12.5 μL 2× EasyTaq PCR SuperMix (TransGen Biotech, Beijing, China), 0.5 μL of each primer and 1 μL of DNA template diluted to a final volume of 25 μL. PCR reactions were performed on a MyCycler Thermal Cycler (Bio-Rad). Amplification was always initiated by placing the PCR tubes into the preheated (94°C) thermal block of the thermocycler. The thermal profile for bacterial genes amplification was as follows: 6 min at 95°C; 20 cycles of 1 min at 94°C, 45 s at 65°C, and 45 s at 72°C; 15 cycles of 1 min at 94°C, 45 s at 55°C, and 45 s at 72°C; 10 min at 72°C. That of fungus genes was 5 min at 94°C; 10 cycles of 30 s at 94°C, 1 min at 56°C, and 1 min at 72°C; 25 cycles of 30 s at 94°C, 1 min at 52°C, and 1 min at 72°C; 10 min at 72°C. All PCR products were electrophoresed on 1% agarose to verify their size and quality. PCR products with the correct size and similar yields (~100 ng μL^-1^) were used for DGGE analysis.

The PCR amplicons were separated by DGGE using a D-Code universal mutation detection system (Bio-Rad, USA) according to the manufacturer’s instructions. Briefly, 20 μL, corresponding to approximately 2000 ng DNA, of each PCR product was loaded onto an 8% (wt/vol) polyacrylamide gel (acrylamide: bisacrylamide = 37.5:1) with a denaturant gradient of 45%-65% for bacteria and 25%-55% for fungus (100% denaturant contains 7 M urea and 40% deionized formamide). Electrophoresis was then conducted at 60°C in 1× tris-acetate-EDTA buffer at 75 V for 16 h. After DGGE, the gels were stained with 1:10,000 SYBR green I for 30 min and then scanned with a Bio-Rad image scanner. Band intensity and position data were analyzed using Quantity One (Bio-Rad, USA).

### Statistical analysis

For each variable measured in the soil, the data were analyzed by one-way ANOVA with SAS 9.1 using Fisher's least significant differences (LSD, *p* = 0.05) to determine significant differences among treatment means. Principal component analysis (PCA) was carried out with Canoco for Windows (version 4.5) software and drawn by Adobe Illustrator CS4. Enzyme activities were calculated by Origin 8 and MS Excel 2003, respectively. Clustering analysis of DGGE patterns were carried out based on the method of UPGMA with Quantity One. Redundancy analysis (RDA) with the Monte Carlo permutation test (499 permutations) was performed to determine if soil microbial community composition and structure were correlated with variation on enzyme activities and physic-chemical parameters, as implemented in Canoco for Windows version 4.5. Some other complemental calculations were carried out using MS Excel 2010. Simple correlation analysis was applied to detect the relations among soil pH, nutrient concentrations and enzyme activities in soils that were treated with different fertilization.

## Results

### Rice yield and albic soil nutrient concentration

The data on rice yield and albic soil physicochemical parameters were given in Table 2. The results showed that the NPK+M and NPK+S treatments significantly increased the rice yield and the yield of NPK+M treatment was approximately 24% greater than of NPK treatment. In general, soil pH ranged from 5.12 to 5.50 and was significantly decreased with organic fertilizers (NPK+M, NPK+S, NPK+G treatments). Compared with the NPK treatment, the additions of different organic manures had different improving effects on SOC, total N, NH_4_^+^-N, available P and K levels. The NPK+M treatment increased SOC and available P; the NPK+S treatment significantly increased SOC and available K and green manure (NPK+G) significantly improved total N, NH_4_^+^-N and available P.

**Table 2:** Rice yield and albic paddy soil pH, SOC and levels of N, P, K in soils under different organic manure amendments.

Treatments	Yield	pH	SOC	Total N	NO_3_^-^-N	NH_4_^+^-N	Available P	Available K
	(kg/ha)		(g/kg)	(g/kg)	(mg/kg)	(mg/kg)	(mg/kg)	(mg/kg)
NPK	6280.0±132.8c	5.50±0.05a	8.77±1.23b	0.76±0.01b	0.17±0.02a	13.14±0.13b	9.24±0.73b	103.54±2.40b
NPK+M	7777.5±157.6a	5.32±0.02b	12.19±1.08a	0.84±0.04ab	0.14±0.02a	15.59±1.25ab	14.67±1.07a	117.08±1.56b
NPK+S	6756.3±65.7b	5.28±0.02b	13.63±1.18a	0.86±0.03ab	0.15±0.02a	15.73±1.98ab	11.31±1.71ab	142.28±7.02a
NPK+G	6293.8±103.1c	5.12±0.03c	11.18±0.81ab	0.89±0.02a	0.17±0.05a	19.05±0.64a	14.36±1.19a	104.94±1.19b

Data are means ± standard error, n = 3. Different letters indicate significant differences among fertilizer treatments at *p* <0.05 (Fish’s LSD test).

### Enzyme activities

**Fig 1:**
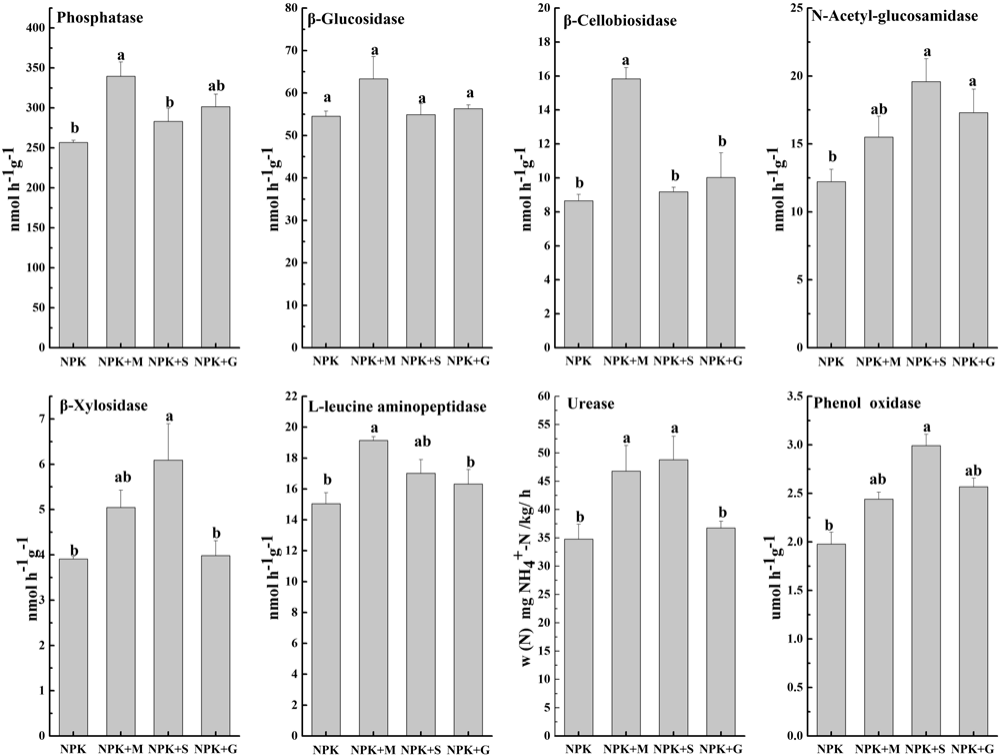
Effects of different organic manures on the activities of extracellular enzymes. Bars indicate standard error, n = 3. Different letters indicate significantly different means at *p* <0.05 (Fish’s LSD test).

The activities of eight extracellular enzymes were quantified in different fertilizer treatments. There were changes in enzyme activities under the different organic treatments, but not in the same way (Fig 1). The NPK+M treatment significantly enhanced the activities of phosphatase, β-cellobiosidase, L-leucine aminopeptidase and urease and the NPK+S treatment significantly enhanced the activities of N-acetyl-glucosamidase, β-xylosidase, urease and phenol oxidase to a significant level. The β-glucosidase activity in NPK+M was higher compared to the other treatments, but did not reach a significant level.

### PLFA analysis

#### Soil microbial biomass

A total of 60 PLFAs were identified and used as measures of total microbial biomass. Abundance of microbial groups for data analysis to determine the microbial community composition under various fertilizer treatments was calculated (Table 3). The amendment of different organic manures significantly increased the total PLFA abundance. The abundance of bacterial, gram-positive, and gram-negative bacterial PLFA were significantly higher in the NPK+S and NPK+M treatments than that of NPK treatment. The fungal PLFA biomass under NPK+S was significantly higher than NPK and NPK+G treatments. G+/G- ratio was significantly increased under NPK+S than the other organic treatments. However, there was no obvious difference of fungi/bacteria ratio under the four treatments.

**Table 3:** Amounts of total, bacterial, fungal, actinomycic, gram-positive bacterial and gram-negative bacterial PLFAs (nmol g^-1^) under different organic manure amendments.

Treatments	Total PLFAs	Bacterial PLFA	Fungal PLFA	Actinomycic PLFA	Gram-positive(G+) PLFA	Gram-negative(G-) PLFA	G+/G-	Fungi/ Bacteria
NPK	33.08±0.11b	21.01±0.27b	3.59±0.25bc	3.45±0.08a	12.82±0.13c	8.19±0.14c	1.57±0.01ab	0.17±0.01a
NPK+M	37.67±0.40a	24.26±0.44a	4.17±0.20ab	3.89±0.20a	14.59±0.32ab	9.66±0.27a	1.51±0.05b	0.17±0.01a
NPK+S	40.74±1.43a	25.29±0.25a	4.28±0.11a	3.93±0.32a	16.02±0.30a	9.27±0.21ab	1.73±0.06a	0.17±0.01a
NPK+G	37.98±1.03a	22.45±0.85b	3.44±0.15c	3.83±0.18a	13.63±0.65bc	8.82±0.21bc	1.54±0.04b	0.15±0.00a

Data are means ± standard error, n = 3. Different letters indicate significant differences among treatments at *p* <0.05 (Fish’s LSD test).

#### Soil microbial structure diversity

**Fig 2:**
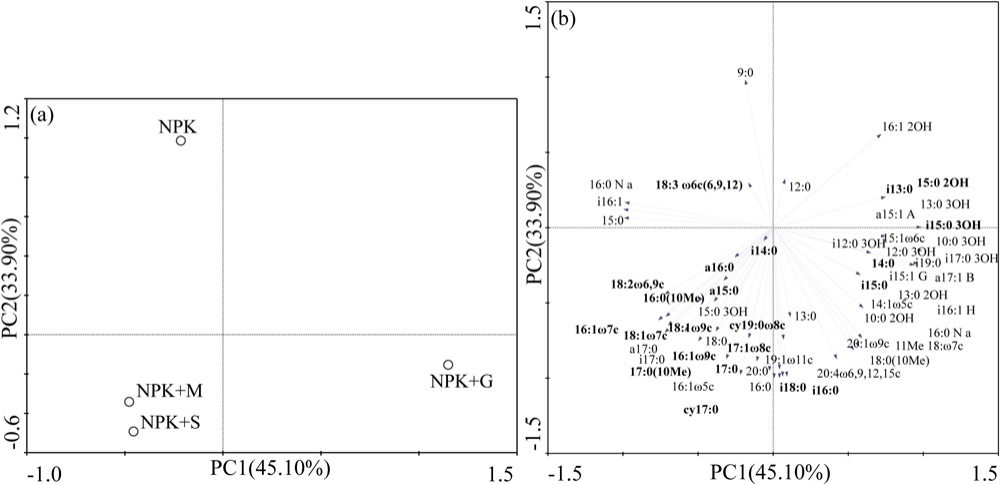
Principal component analysis of the PLFA patterns from soils under different organic manure applications (a) and loadings of the individual PLFAs (b) from the data of principal components 1 and 2. Factors 1 and 2 accounted for 45.10% and 33.90%, respectively, of the variance.

The PLFAs were also used to assess whether the observed changes in microbial composition parameters were accompanied by changes in the composition of microbial communities under different organic manure additions. Differences were identified using PCA and outlined according to proportions of structural classes, biomarkers and individual fatty acids present under each treatment. The first two principal components (PCs) accounted for 45.10% and 33.90%, respectively, of the overall variance, and the PLFA profiles showed a significant separation when comparing soil samples from NPK+G to the other treatments along the PC1 axis (Fig 2a). The dominant PLFAs located to the right along PC1 were i13:0, 14:0, i15:0, 15:0 2OH and i15:0 3OH (Fig 2b). A clear separation was also found when comparing the PLFA patterns of soil samples from the NPK treatment to the other organic amendments along the PC2 axis. Conversely, the dominant PLFAs 17:0, i18:0, 16:0(10Me), 17:0(10Me), cy17:0 and cy19:0ω8c were negatively correlated with PC2.

Particular identified PLFAs, including branched i14:0, a15:0 and a16:0, monounsaturated 18:1ω9c, 18:1ω7c, 16:1ω9c, 16:1ω7c, 16:1ω5c, 17:1ω8c, 18:2ω6,9c and cyclopropane cy17:0, cy19:0ω8c fatty acids were on the left side of the plot, while most of the saturated straight chain lipids (i13:0, 14:0, i15:0, i16:0, i18:0) were on the right. This indicates that soils with the NPK+M and NPK+S treatments contained higher proportions of unsaturated and cyclopropane fatty acids, most of which are biomarkers of fungi and gram-negative bacteria, while straight chain saturated fatty acids, which mostly represent gram-positive bacteria, were more abundant in the NPK+G treatment.

### PCR-DGGE analysis

**Fig 3:**
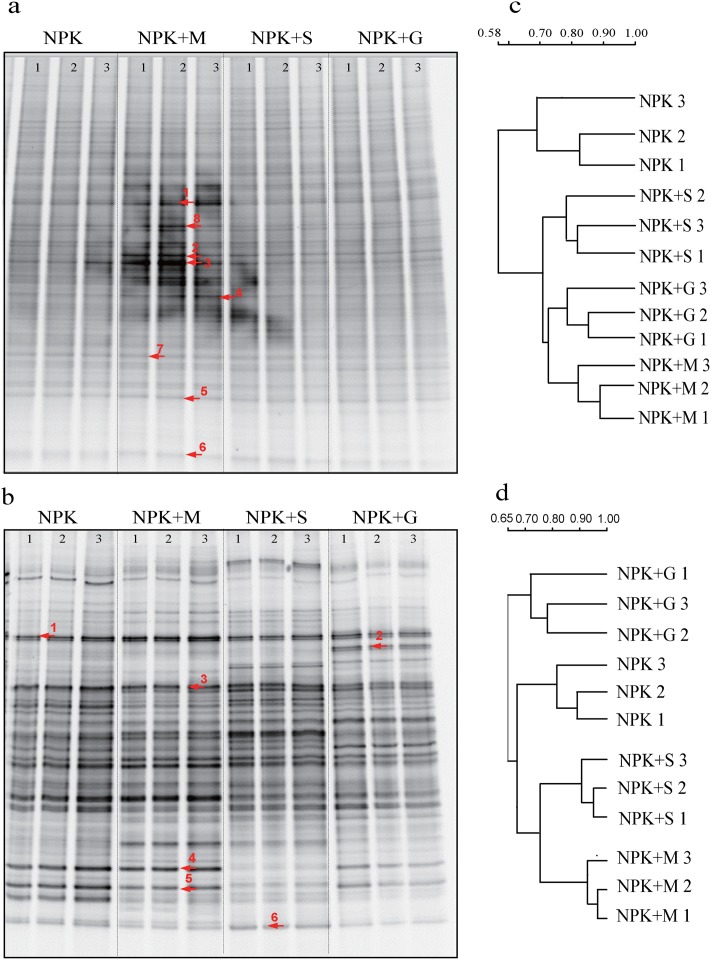
DGGE banding patterns of soil bacteria (a) and fungi (b) and cluster analyses of bacteria (c) and fungi (d) under different fertilizer treatments.

**Fig 4:**
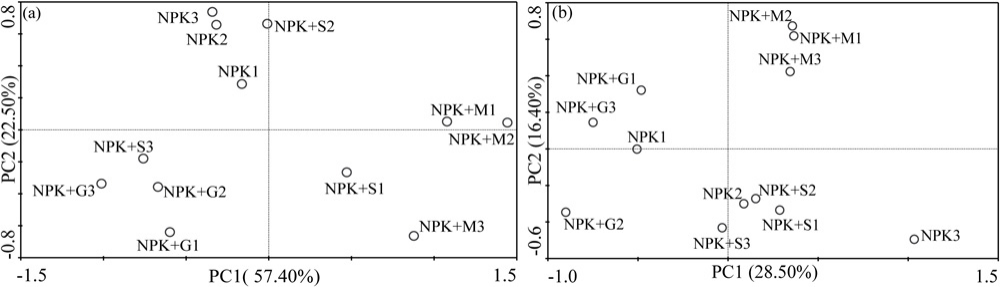
Principal component analyses of bacterial (a) and fungal (b) DGGE banding patterns of different treatments.

The bacterial community structure determined by the DGGE banding patterns of partial 16S rDNA amplified with primers GC-357f and 518r and the fungal community structure determined by the DGGE banding patterns of partial 18S rDNA amplified with primers ITS1-F and ITS2 are shown in Fig 3a and Fig 3b. The DGGE patterns from different soil samples were generally similar, although several strong bands were observed in individual treatments, such as those marked with red arrows. It was especially obvious in bands 1–7 in Fig 3a which were added in the NPK+M treatment when compared with the other treatments. This indicated that the addition of livestock manure enhanced the bacterial biomass of some species and thus affected the microbial community structure. Meanwhile, several fungal community bands emerged or were missing in the NPK+G treatment (Fig 3b). This may indicate that the mechanism of the green manure impact on fugal community was different from the other treatments. It was also confirmed by the cluster analysis of DGGE banding pattern (Fig 3d) in which the NPK+G treatment was distinctly classified. Accompanied with the PCA results of the bacterial and fungal DGGE banding patterns (Fig 4), treatments were relatively better separated along PC1 and PC2 in Fig 4b than that in Fig 4a. It confirmed that different organic manures had a greater influence on fungal than bacterial community.

### Correlations

No significant relationship between biochemical properties and soil pH was observed, while total N and available P were negatively related to soil pH values (Table 4) in this experiment. Stronger correlations were detected between SOC and β-xylosidase, phenol oxidase and bacterial PLFA abundance. Furthermore, significant correlations were detected between some of the tested enzymes. For example, activities of phosphatase and β-cellobiosidase were significantly correlated with an *r* value of 0.77**.

**Table 4:** Correlation coefficients (r) for simple correlation analysis between soil pH, nutrient concentration, enzyme activities and PLFA profiles under different fertilizer treatments.

	pH	SOC	TN	AP	AK	Pho	βG	NAG	βX	Urease	PhOx	Bacterial PLFA	Fungal PLFA
pH													
SOC													
Total N	-0.71**												
AP	-0.60*		0.74**										
AK		0.63*											
Pho				0.85**									
βG													
βCB				0.63*		0.77**	0.61*						
NAG-					0.69*								
βX		0.68*			0.61*								
LAP				0.69*		0.71**							
Urease					0.60*								
PhOx		0.64*			0.76**			0.72**					
Bacterial PLFA		0.74**			0.76**				0.71*	0.76**	0.59*		
Fungal PLFA					0.58*				0.73**			0.63*	
Actinomycic PLFA													
G+/G-			-0.59*		0.73*						0.71*		
F/B			-0.65*										0.74**

Note: Abbreviations: TN total N, AP available P, AK available K, Pho phosphatase, βG β-glucosidase, βCB β-cellobiosidase, NAG N-acetyl-glucosaminidase, βX β-xylosidase, LAP L-leucine aminopeptidase, PhOx phenol oxidase.

*Significant at *p* <0.05;

**significant at *p* <0.01.

**Fig 5:**
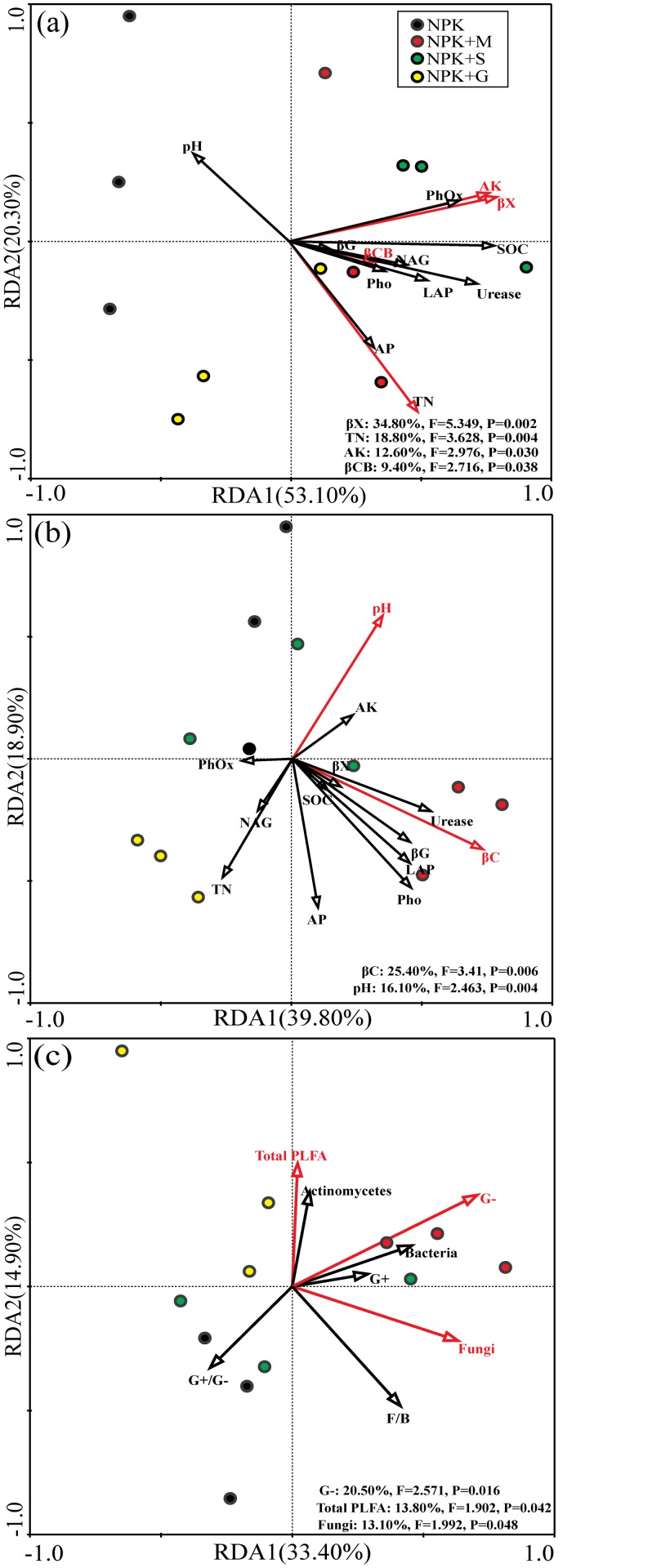
Redundancy analyses (RDA) of the correlations between soil physicochemical properties and PLFA parameters (a), the correlations between soil physicochemical properties and DGGE banding patterns (b) and the correlations between PLFA parameters and DGGE banding patterns (c). The red arrows indicate the soil parameters that had strong and significant impact on enzyme activities (*p* <0.05), and corresponding explained proportion of variability was shown in the lower right corner.

The redundancy analysis (RDA) was carried out for soil properties and enzyme activities with PLFA parameters of the four treatments. Soil properties and enzyme activities were used as environmental variables (Fig 5a). The first and second axes accounted for 53.10% and 20.30% of the total variation between soil physicochemical and biochemical properties and microbial community composition assessed by PLFAs. Microbial community composition were significant correlated with β-xylosidase (*F* = 5.349, *P* = 0.002), β-cellobiosidase (*F* = 2.716, *P* = 0.038) activities, total N (*F* = 3.628, *P* = 0.004) and available K (*F* = 2.976, *P* = 0.030) contents.

Results of RDA analysis between soil properties and enzyme activities with microbial community structure as indicated by DGGE banding patterns are shown in Fig 5b. The first and second axes accounted for 39.80% and 18.90%, respectively, of the total variation in microbial community structure. The microbial community structure was significantly correlated with β-cellobiosidase (*F* = 3.41, *P* = 0.006) and pH (*F* = 2.463, *P* = 0.004).

The RDA showed the relationships of PLFA parameters with DGGE banding patterns (Fig 5c), and the first and second axes accounted for 33.40% and 14.90%, respectively, of the total variation. Gram-negative (*F* = 2.571, *P* = 0.016), total PLFA (*F* = 1.902, *P* = 0.042) and fungi (*F* = 1.992, *P* = 0.048) which explained 20.50%, 13.80% and 13.10% of the total variability in the changes of microbial community composition and structure.

## Discussion

It is commonly acknowledged that paddy soils with organic fertilization can maintain soil sustainability and enhance more accumulation of organic C than arable soil relying on chemical fertilizer only [4, 52, 53]. The results of this study indicated that, compared to chemical fertilizer, organic amendments improved rice yield and physic-chemical character of albic paddy soil which also significantly enhanced enzyme activities and altered microbial community at the same time. The improving impacts that different organic manures had on different parameters of soil fertility might be a consequence of their relative dominant component, for example, there was a relatively higher level of K in straw and a relatively higher level of P in livestock manure among the three organic manures which may consequently result in the obvious increase of available P under NPK+M and significant increase of K concentration under NPK+S treatment. Organic fertilizers are believed to stabilize or even increase soil pH [11, 54], which we did not observe in this experiment. Although no significant correlation was detected between bacterial communities with soil pH under a typical clay loamy anthrosol [32], many studies have reported that environmental factors altered soil microbial community, especially the soil pH, which has been demonstrated in several studies to be the strongest factor shaping microbial community structure [55, 56, 57]. This was confirmed by RDA analysis (Fig 5b) that DGGE banding patterns were significantly related to soil pH (*F* = 2.463, *P* = 0.004), but we did not find significant relationship between enzyme activities and soil pH (Table 4). This is different from Deng et al. [54] and Shen et al. [57] who reported enzyme activities and biotransformation processes in soil to be closely associated with pH values. The soil pH values varied significantly from 3 to 9 in those studies allowing insight into the relationship between pH and soil bacterial communities [55, 58, 59]. Thus, we hypothesize that there are important factors other than pH in shaping soil bacterial communities. As reported by Navarrete et al. [60], abiotic soil factors (not only related to soil acidity) such as Al, Ca, Mg, Mn and B could also drive the acidobacterial populations. In this study, RDA analysis of soil properties and enzyme activities with PLFA parameters (Fig 5a) showed that changes of microbial community composition mostly depended on β-xylosidase (*F* = 5.349, *P* = 0.002), β-cellobiosidase (*F* = 2.716, *P* = 0.038) activities, total N (*F* = 3.628, *P* = 0.004) and available K (*F* = 2.976, *P* = 0.030) contents, which explained accumulatively 75.60% of the total variance. As enzyme and microbial activities were significantly correlated with SOC as reported by other studies [17, 61], stronger correlations were also detected between SOC and β-xylosidase, phenol oxidase and bacterial PLFA abundance (Table 4) in this study. Therefore, the soil pH may not represent an integrating variable in our study due to the mild variation of soil pH, which suggests that soil characteristics such as soil nutrients, rather than soil pH can shape the microbial community.

Furthermore, organic manures increased biological activity [62]. In this experiment, three organic manures all had positive effects on the enzyme activity of albic paddy soil. Ramesh et al. [63] reported that the enhanced level of soil enzyme activity due to addition of organic manures promotes the recycling of nutrients in the soil ecosystem. Bowen and Harper [64] also reported that on one hand, straw increased the soil microbial biomass and improved the activity of microorganisms but on the other hand, the increase in soil microbial biomass and microbial activity also accelerated the decay of the straw which brought more nutrients to the soil. The β-cellobiosidase is involved in the biological degradation of cellulose, and can be induced by cellulose or straw, but in this study, treatment NPK+S did not show higher activity of this enzyme. Previous studies showed that cellulose contained in straw usually finished decomposing just at the beginning of the straw returning [65, 66, 67] and the β-cellobiosidase activity showed a trend of rapidly increased activity and relatively stable activity at first, but tended to significantly decrease after 60 days of straw returning [65]. This may explain the relatively lower β-cellobiosidase activity of NPK+S treatment during the harvest period.

Rice yield was enhanced in the treatments where only chemical fertilizer was added [61], but the organic amendments generally had more effects on most of the paddy microbial indicators than chemical fertilizer only [22], which was convinced by the obvious changes of PLFA abundance of microbial groups in this study. As Böhme [68] reported, the soil microbial biomass was fundamental to maintain soil functions because it represented the main source of soil enzymes which regulated the transformation processes of elements in soils. The highest PLFA biomass detected in the straw treatment was also associated with higher organic C and extracellular enzyme activities involved in C, N and P mineralization. This phenomenon may be due to the fact that straw addition increased the amount of available energy in albic paddy soil during the subsequent incubation and thus led to remarkable changes in the microbial biomass [69]. Stark et al. [15] also reported soil management systems that conserved or improved organic matter content ultimately enhanced the soil microbial biomass and activity.

Despite of the improving effects of organic manures, differences in organic matter composition and thus substrate availability are likely to be the reason for the differences in microbial community structure. The NPK+M and NPK+S treatments increased the PLFA biomarkers for bacteria, gram-positive and gram-negative bacteria. While the NPK+S treatment significantly increased the fungal PLFA biomass. In general, a lower gram-positive/gram-negative bacteria ratio is indicative of better soil nutrition [70]. A higher fungi/bacteria ratio can reflect the relative abundance of the microbial population which is an important indicator of a stronger soil ecosystem buffering capacity and more sustainable land use [19, 71]. Several studies have shown gram-negative bacteria, which are sensitive to oligotrophic conditions [72], are often stimulated by added organic matter resulting in a low gram-positive/gram-negative bacteria ratio [73, 74]. From the perspective of these two parameters, NPK+M treatment performed better among these four treatments with both a relatively lower G+:G- and a relatively higher fungi/bacteria.

Generally, paddy soils are dominated by actinomycetes and gram-positive bacteria [75]. However, SOC is the key determinant of shifts in microbial communities [76]. Bossio and Scow [23] observed higher levels of 16:1ω5c in plots receiving organic addition compared to inorganic fertilizer. However, in this experiment, 16:1ω7c but not 16:1ω5c (although both are typical of gram-negative bacteria) was present at high levels in soils receiving organic manure except for the green manure treatment in which few of both were detected. Also, there were 52 detected PLFA species in the soils treated with green manure compared with 35 in the chemical treatment. This indicates that organic amendments significantly improved the soil fertility status, which enhanced the microbial diversity, biomass and activity [15]. The addition of different organic manures resulted in different microbial community structure. Soils in the NPK+M and NPK+S treatments contained higher proportions of unsaturated and cyclopropane fatty acids, most of which were biomarkers of fungi and gram-negative bacteria, while straight chain saturated fatty acids that mostly represented gram-positive bacteria existed more in the NPK+G treatment.

The DGGE banding pattern of fungi indicated that with straw or livestock manure addition, the fungal community showed a difference from that under green manure addition. Moreover, fungal species are the first colonizers on soil incorporated straw [64], but they are sensitive to dry conditions. Chu et al. [29], using phylogenetic analysis of DGGE patterns, showed that the change in the bacterial community in organic manure fertilized soil might not be because of the direct influence of the bacteria in the compost, but because of the promoting effect of the compost on the growth of an indigenous Bacillus sp. in the soil. However, it is essential to detect whether the differences observed were caused by the difference in native microorganisms in the organic manures or the priming effects that the organic manures stimulated. The fungal phylogenetic tree analysis showed that the NPK+G treatment was clearly classified which was also confirmed by the obvious relative lower fungal PLFA biomass compared to the other treatments. In our study, the abundances of gram-negative bacteria and fungi significantly affected genetic diversity of microorganism (Fig 5c), suggesting that these microbial groups were probably effective in improving fertility of low-yield albic paddy soil.

## Conclusion

The productivity of rice was significantly higher with livestock manure and straw addition. Different organic manures could enhance albic paddy soil nutrients, enzyme activities and affect the microbial biomass and structure. Both PLFA and DGGE profiles indicated livestock manure and straw affected biochemical and microbial characteristics of albic paddy soil in a similar way. The addition of livestock manure or straw increased the microbial biomass and had positive effects on soil fertility conditions and the ecological buffering capacity, which contained higher proportions of unsaturated and cyclopropane fatty acids, most of which are biomarkers of fungi and gram-negative bacteria. Straight chain saturated fatty acids that mostly represent gram-positive bacteria were more abundant in the green manure treatment. The PCA analysis of DGGE patterns showed that organic amendments had a greater influence on the fungi than in the bacterial community. Meanwhile, the effect of NPK+M on the bacterial community was more obvious. RDA analysis revealed the amounts of gram-negative bacteria and fungi were probably effective in improving fertility of low-yield albic paddy soil because of their significant influence on DGGE profile. These findings implied that different organic manures promoted soil biochemical and microbial characteristics by different means and thus improved albic paddy soil quality.

## References

[1] Mandal A, Patra AK, Singh D, Swarup A, Masto RE. Effect of long-term application of manure and fertilizer on biological and biochemical activities in soil during crop development stages. Bioresource Technol. 2007;98: 3585–3592. 17207997 10.1016/j.biortech.2006.11.027

[2] Wang QJ, Bai YH, Gao HW, He J, Chen H, Chesney RC, et al Soil chemical properties and microbial biomass after 16 years of no-tillage farming on the Loess Plateau, China. Geoderma. 2008;144: 502–508.

[3] Huang QR, Hu F, Huang S, Li HX, Yuan YH, Pan GX, et al Effect of long-term fertilization on organic carbon and nitrogen in a subtropical paddy soil. Pedosphere. 2009;19: 727–734.

[4] Hao XH, Liu SL, Wu JS, Hu RG, Tong CL, Su YY. Effect of long-term application of inorganic fertilizer and organic amendments on soil organic matter and microbial biomass in three subtropical paddy soils. Nutr Cycl Agroecosys. 2008;81: 17–24.

[5] Gil-Sotres F, Trasar-Cepeda C, Leirós MC, Seoane S. Different approaches to evaluating soil quality using biochemical properties. Soil Biol Biochem. 2005;37: 877–887.

[6] Dinesh R, Srinivasan V, Hamza S, Manjusha A. Short-term incorporation of organic manures and biofertilizers influences biochemical and microbial characteristics of soils under an annual crop [Turmeric (Curcuma longa L.)]. Bioresource Technol. 2010;101: 4697–4702. 10.1016/j.biortech.2010.01.108 20163953

[7] Rashid MI, de Goede RG, Brussaard L, Lantinga EA. Homefield advantage of cattle manure decomposition affects the apparent nitrogen recovery in production grasslands. Soil Biol Biochem. 2013;57: 320–326.

[8] Allison SD, Jastrow JD. Activities of extracellular enzymes in physically isolated fractions of restored grassland soils. Soil Biol Biochem. 2006;38: 3245–3256.

[9] Nannipieri P, Giagnoni L, Renella G, Puglisi E, Ceccanti B, Masciandaro G, et al Soil enzymology: classical and molecular approaches. Biol Fertil Soils. 2012;48: 743–762.

[10] Mele PM, Crowley DE. Application of self-organizing maps for assessing soil biological quality. Agric Ecosyst Environ. 2008;126: 139–152.

[11] Zhong WH, Gu T, Wang W, Zhang B, Lin XG, Huang QR, et al The effects of mineral fertilizer and organic manure on soil microbial community and diversity. Plant Soil. 2010;326: 511–522.

[12] Kennedy AC, Smith KL. Soil microbial diversity and the sustainability of agricultural soils. Plant Soil. 1995;170: 75–86.

[13] Anderson TH. Microbial eco-physiological indicators to asses soil quality. Agric Ecosyst Environ. 2003;98: 285–293.

[14] Bending GD, Turner MK, Rayns F, Marx MC, Wood M. Microbial and biochemical soil quality indicators and their potential for differentiating areas under contrasting agricultural management regimes. Soil Biol Biochem. 2004;36: 1785–1792.

[15] Stark C, Condron LM, Stewart A, Di HJ, O’Callaghan M. Influence of organic and mineral amendments on microbial soil properties and processes. Appl Soil Ecol. 2007;35: 79–93.

[16] Helgason BL, Walley FL, Germida JJ. No-till soil management increases microbial biomass and alters community profiles in soil aggregates. Appl Soil Ecol. 2010;46: 390–397.

[17] Ai C, Liang GQ, Sun JW, Wang XB, Zhou W. Responses of extracellular enzyme activities and microbial community in both the rhizosphere and bulk soil to long-term fertilization practices in a fluvo-aquic soil. Geoderma. 2012;173: 330–338.

[18] De Vries FT, Manning P, Tallowin JRB, Mortimer SR, Pilgrim ES, Harrison KA, et al Abiotic drivers and plant traits explain landscape-scale patterns in soil microbial communities. Ecol Lett. 2012;15: 1230–1239. 10.1111/j.1461-0248.2012.01844.x 22882451

[19] Bossio DA, Scow KM. Impacts of carbon and flooding on soil microbial communities: phospholipid fatty acid profiles and substrate utilization patterns. Microb Ecol. 1998;35: 265–278. 9569284 10.1007/s002489900082

[20] Ling N, Sun YM, Ma JH, Guo JJ, Zhu P, Peng C, et al Response of the bacterial diversity and soil enzyme activity in particle-size fractions of Mollisol after different fertilization in a long-term experiment. Biol Fert Soils. 2014;50: 901–911.

[21] Marschner P, Kandeler E, Marschner B. Structure and function of the soil microbial community in a long-term fertilizer experiment. Soil Biol Biochem. 2003;35: 453–461.

[22] Liu MQ, Hu F, Chen XY, Huang QR, Jiao JG, Zhang B, et al Organic amendments with reduced chemical fertilizer promote soil microbial development and nutrient availability in a subtropical paddy field: The influence of quantity, type and application time of organic amendments. Appl Soil Ecol. 2009;42: 166–175.

[23] Bossio DA, Scow KM, Gunapala N, Graham KJ. Determinants of soil microbial communities: effects of agricultural management, season, and soil type on phospholipid fatty acid profiles. Microb Ecol. 1998;36: 1–12. 9622559 10.1007/s002489900087

[24] Moeskops B, Buchan D, Sleutel S, Herawaty L, Husen E, Saraswati R, et al Soil microbial communities and activities underintensive organic and conventional vegetable farming in West Java, Indonesia. Appl Soil Ecol. 2010;45: 112–120.

[25] Moeskops B, Buchan D, Van Beneden S, Fievez V, Sleutel S, Gasper MS, et al The impact of exogenous organic matter on SOM contents and microbial soil quality. Pedobiologia. 2012;55: 175–184.

[26] Muyzer G. DGGE/TGGE: A method for identifying genes from natural ecosystems. Current Opinion Microbiology. 1999;2: 317–322. 10383868 10.1016/S1369-5274(99)80055-1

[27] Nakatsu CH, Torsvik V, Øvreås L. Soil community analysis using DGGE of 16S rDNA polymerase chain reaction products. Soil Sci Soc Am J. 2000;64: 1382–1388.

[28] Yao H, He ZL, Wilson MJ, Campbell CD. Microbial biomass and community structure in a sequence of soils with increasing fertility and changing land use. Microb Ecol. 2000;40: 223–237. 11080380 10.1007/s002480000053

[29] Chu HY, Lin XG, Fujii T, Morimoto S, Yagi K, Hu JL, et al Soil microbial biomass, dehydrogenase activity, bacterial community structure in response to long-term fertilizer management. Soil Biol Biochem. 2007;39: 2971–2976.

[30] Jin G, Kelly R. Characterization of microbial communities in a pilot-scale constructed wetland using PLFA and PCR-DGGE analyses. Journal of Environmental Science and Health Part A. 2007;42: 1639–1647. 17849306 10.1080/10934520701518125

[31] Gil SV, Meriles J, Conforto C, Basanta M, Radl V, Hagn A, et al Response of soil microbial communities to different management practices in surface soils of a soybean agroecosystem in Argentina. Eur J Soil Bio. 2011;47: 55–60.

[32] Zhao J, Ni T, Li Y, Xiong W, Ran W, Shen B, et al Responses of bacterial communities in arable soils in a rice-wheat cropping system to different fertilizer regimes and sampling times. PLOS ONE. 2014;9: e85301 10.1371/journal.pone.0085301 24465530 PMC3896389

[33] Yu QY, Xiong GT. Clay soil fertility status and its improved utilization. Journal of Anhui Agricultural Sciences. 2004;32: 1161–1162.

[34] Zhong WH, Cai ZC. Long-term effects of inorganic fertilizers on microbial biomass and community functional diversity in a paddy soil derived from quaternary red clay. Appl Soil Ecol. 2007;36: 84–91.

[35] Zhong WH, Cai ZC, Zhang H. Effects of long-term application of inorganic fertilizers on biochemical properties of a rice-planting red soil. Pedosphere. 2007;17: 419–428.

[36] Nelson PN, Oades JM. Organic matter, sodicity, and soil structure In: Sumner ME, Naidu R, editors. Sodic Soils. New York: Oxford University Press; 1998 pp. 51–75.

[37] Kalembasa SJ, Jenkinson DS. A comparative study of titrimetric and gravimetric methods for the determination of organic carbon in soil. Journal of the Science of Food and Agriculture. 1973;24: 1085–1090.

[38] Bremner JM, Mulvaney CS. Nitrogen—total In: Page AL, Miller RH, Keeney DR, editors. Methods of soil analysis, Part 2. Chemical and microbiological properties. Madison, WI: ASA; 1982 pp. 643–698.

[39] Horwath WR, Paul EA. Microbial biomass In: Weaver RW, Angle JS, Bottomley PS, editors. Methods of soil analysis, Part 2. Microbiological and biochemical properties. Madison, WI: SSSA; 1994 pp. 753–773.

[40] Olsen SR, Sommers LE. Phosphorus In: Page AL, Miller RH, Keeney DR, editors. Methods of soil analysis. Part 2. Chemical and microbiological properties. Madison, WI: SSSA; 1982 pp. 403–430.

[41] DeForest J. The influence of time, storage temperature, and substrate age on potential soil enzyme activity in acidic forest soils using MUB-linked substrates and L-DOPA. Soil Biol Biochem. 2009;41: 1180–1186.

[42] Saiya—Cork KR, Sinsabaugh RL, Zak DR. The effects of long term nitrogen deposition on extracellular enzyme activity in an Acer saccharum forest soil. Soil Biol Biochem. 2002;34: 1309–1315.

[43] Wittmann C, Kähkönen MA, Ilvesniemi H, Kurola J, Salkinoja—Salonen MS. Areal activities and stratification of hydrolytic enzymes involved in the biochemical cycles of carbon, nitrogen, sulphur and phosphorus in podsolized boreal forest soils. Soil Biol Biochem. 2004;36: 425–433.

[44] Lu RK. Analytical methods of soil and agro-chemistry (in Chinese). Beijing, China: Agricultural Science and Technology Press; 2000.

[45] Wu YP, Ding N, Wang G, Xu JM, Wu JJ, Brookes PC. Effects of different soil weights, storage times and extraction methods on soil phospholipid fatty acid analyses. Geoderma. 2009;150: 171–178.

[46] Frostegrd A, Bååth E, Tunlio A. Shifts in the structure of soil microbial communities in limed forests as revealed by phospholipid fatty acid analysis. Soil Biol Biochem. 1993;25: 723–730.

[47] Green C, Scow K. Analysis of phospholipid fatty acids (PLFA) to characterize microbial communities in aquifers. Hydrogeology Journal. 2000;8: 126–141.

[48] Ai C, Liang GQ, Sun JW, He P, Tang SH, Yang SH, et al The alleviation of acid soil stress in rice by inorganic or organic ameliorants is associated with changes in soil enzyme activity and microbial community composition. Biol Fert Soils. 2015; 1–13.

[49] Hill GT, Mitkowski NA, Aldrich—Wolfe L, Emele LR, Jurkonie DD, Ficke A, et al Methods for assessing the composition and diversity of soil microbial communities. Appl Soil Ecol. 2000;15: 25–36.

[50] Anderson IC, Campbell CD, Prosser JI. Diversity of fungi in organic soils under a moorland—Scots pine (Pinus sylvestris L.) gradient. Environ Microbiol. 2003;5: 1121–1132. 14641592 10.1046/j.1462-2920.2003.00522.x

[51] Caliz J, Vila X, Martí E, Sierra J, Cruañas R, Garau MA, et al Impact of chlorophenols on microbiota of an unpolluted acidic soil: microbial resistance and biodegradation. FEMS Microbiol Ecol. 2011;78: 150–164. 10.1111/j.1574-6941.2011.01093.x 21426365

[52] Lal R. Soil carbon sequestration impacts on global climate change and food security. Science. 2004;304: 1623–1627. 15192216 10.1126/science.1097396

[53] Pan G, Li L, Wu LS, Zhang X. Storage and sequestration potential of topsoil organic carbon in China’s paddy soils. Global Change Biol. 2004;10: 79–92.

[54] Deng SP, Parham JA, Hattey JA, Babu D. Animal manure and anhydrous ammonia amendment alter microbial carbon use efficiency, microbial biomass, and activities of dehydrogenase and amidohydrolases in semiarid agroecosystems. Appl Soil Ecol. 2006;33: 258–268.

[55] Rousk J, Bååth E, Brookes PC, Lauber CL, Lozupone C, Caporaso JG, et al Soil bacterial and fungal communities across a pH gradient in an arable soil. The ISME J. 2010;4: 1340–1351. 10.1038/ismej.2010.58 20445636

[56] Lauber CL, Hamady M, Knight R, Fierer N. Pyrosequencing-based assessment of soil pH as a predictor of soil bacterial community structure at the continental scale. Appl Environ Microbiol. 2009;75: 5111–5120. 10.1128/AEM.00335-09 19502440 PMC2725504

[57] Shen CC, Xiong JB, Zhang HY, Feng YZ, Lin XG, Li XY, et al Soil pH drives the spatial distribution of bacterial communities along elevation on Changbai Mountain. Soil Biol Biochem. 2012;57:204–211.

[58] Chu H, Fierer N, Lauber CL, Caporaso J, Knight R, Grogan P. Soil bacterial diversity in the Arctic is not fundamentally different from that found in other biomes. Environ Microbiol. 2010;12: 2998–3006. 10.1111/j.1462-2920.2010.02277.x 20561020

[59] Jones RT, Robeson MS, Lauber CL, Hamady M, Knight R, Fierer N. A comprehensive survey of soil acidobacterial diversity using pyrosequencing and clone library analyses. The ISME J. 2009;3: 442–453. 10.1038/ismej.2008.127 19129864 PMC2997719

[60] Navarrete AA, Kuramae EE, Hollander M, Pijl AS, Veen JA, Tsai SM. Acidobacterial community responses to agricultural management of soybean in Amazon forest soils. FEMS Microbiol Ecol. 2012;83: 607–621. 10.1111/1574-6941.12018 23013447

[61] Stark CH, Condron LM, O’Callaghan M, Stewart A, Di HJ. Differences in soil enzyme activities, microbial community structure and short-term nitrogen mineralisation resulting from farm management history and organic matter amendments. Soil Biol Biochem. 2008;40: 1352–1363.

[62] Barzegar AR, Yousefi A, Daryashenas A. The effect of addition of different amounts and types of organic materials on soil physical properties and yield of wheat. Plant Soil. 2002;247: 295–301.

[63] Ramesh P, Singh M, Panwar NR, Singh AB, Ramana S. Response of pigeonpea varieties to organic manures and their influence on fertility and enzyme activity of soil. Indian Journal of Agricultural Sciences. 2006;76: 252–254.

[64] Bowen RM, Harper SHT. Decomposition of wheat straw and related compounds by fungi isolated from straw in arable soils. Soil Biol Biochem. 1990;22: 393–399.

[65] Christensen BT. Barley straw decomposition under field conditions: effect of placement and initial nitrogen content on weight loss and nitrogen dynamics. Soil Biol and Biochem. 1986;18: 523–529.

[66] Li FY, Sun XF, Feng WQ, Qin YS, Wang CQ, Tu SH. Nutrient release patterns and decomposing rates of wheat and rapeseed straw. Plant Nutrition and Fertilizer Science. 2009;15: 374–380.

[67] Dai ZG, Lu JW, Li XK, Lu MX, Yang WB, Gao XZ. Nutrient release characteristic of different crop straws manure. Transactions of the Chinese Society of Agricultural Engineering. 2010;26: 272–276.

[68] Böhme L, Böhme F. Soil microbiological and biochemical properties affected by plant growth and different long-term fertilization. Eur J Soil Bio. 2006;42: 1–12.

[69] Potthoffa M, Joergensenb RG, Wolters V. Short-term effects of earthworm activity and straw amendment on the microbial C and N turnover in a remoistened arable soil after summer drought. Soil Biol Biochem. 2001;33: 583–591.

[70] Rajendran N, Matsuda O, Rajendran R, Urushigawa Y. Comparative description of microbial community structure in surface sediments of eutrophic bays. Marine Pollution Bulletin. 1997;34: 26–33.

[71] De Vries FT, Hoffland E, van Eekeren N, Brussaard L, Bloem J. Fungal/bacterial ratios in grasslands with contrasting nitrogen management. Soil Biol Biochem. 2006;38: 2092–2103. 16899389

[72] Esperschütz J, Buegger F, Winkler JB, Munch JC, Schloter M, Gattinger A. Microbial response to exudates in the rhizosphere of young beech trees (Fagus sylvatica L.) after dormancy. Soil Biol Biochem. 2009;41: 1976–1985.

[73] Buyer JS, Teasdale JR, Roberts DP, Zasada IA, Maul JE. Factors affecting soil microbial community structure in tomato cropping systems. Soil Biol Biochem. 2010;42: 831–841.

[74] Larkin R, Honeycutt C, Griffin T. Effect of swine and dairy manure amendments on microbial communities in three soils as influenced by environmental conditions. Biol Fert Soils. 2006;43: 51–61.

[75] Kimura M, Asakawa S. Comparison of community structures of microbiota at main habitats in rice field ecosystem based on phospholipid fatty acid analysis. Biol Fert Soils. 2006;43: 20–29.

[76] Drenovsky RE, Vo D, Graham KJ, Scow KM. Soil water content and organic carbon availability are major determinants of soil microbial community composition. Microb Ecol. 2004;48: 424–430. 15692862 10.1007/s00248-003-1063-2

